# Supplementary material for: Recent decreases in snow water storage in western North America
Source: Commun Earth Environ. 2023 May 22;4(1):170. doi: 10.1038/s43247-023-00751-3 (PMC11041790; doi:10.1038/s43247-023-00751-3)
Supplement: Supplementary file 1 — Supplementary Information [file 43247_2023_751_MOESM1_ESM.pdf]

Recent decreases in snow water storage in western North America

Supplemental Information

Katherine. E. Hale[1,2], Keith. S. Jennings[3], Keith. N. Musselman[1,2], Ben. Livneh[4,5], Noah. P. Molotch[1,2,6]

[1]Department of Geography, University of Colorado at Boulder, Boulder, CO, USA
[2]Institute of Arctic and Alpine Research, University of Colorado at Boulder, Boulder, CO, USA
[3]Lynker, Boulder, CO, USA
[4]Cooperative Institute for Research in Environmental Science, University of Colorado at Boulder, Boulder, CO, USA
[5]Department of Civil, Environmental, and Architectural Engineering, University of Colorado at Boulder, Boulder, CO, USA
[6]Jet Propulsion Laboratory, California Institute of Technology, Pasadena, CA, USA

Corresponding author: Katherine.E.Hale@colorado.edu

Supplementary Figure 1: Histograms of long-term $\overline{\mathrm{SSI}}$ distribution.

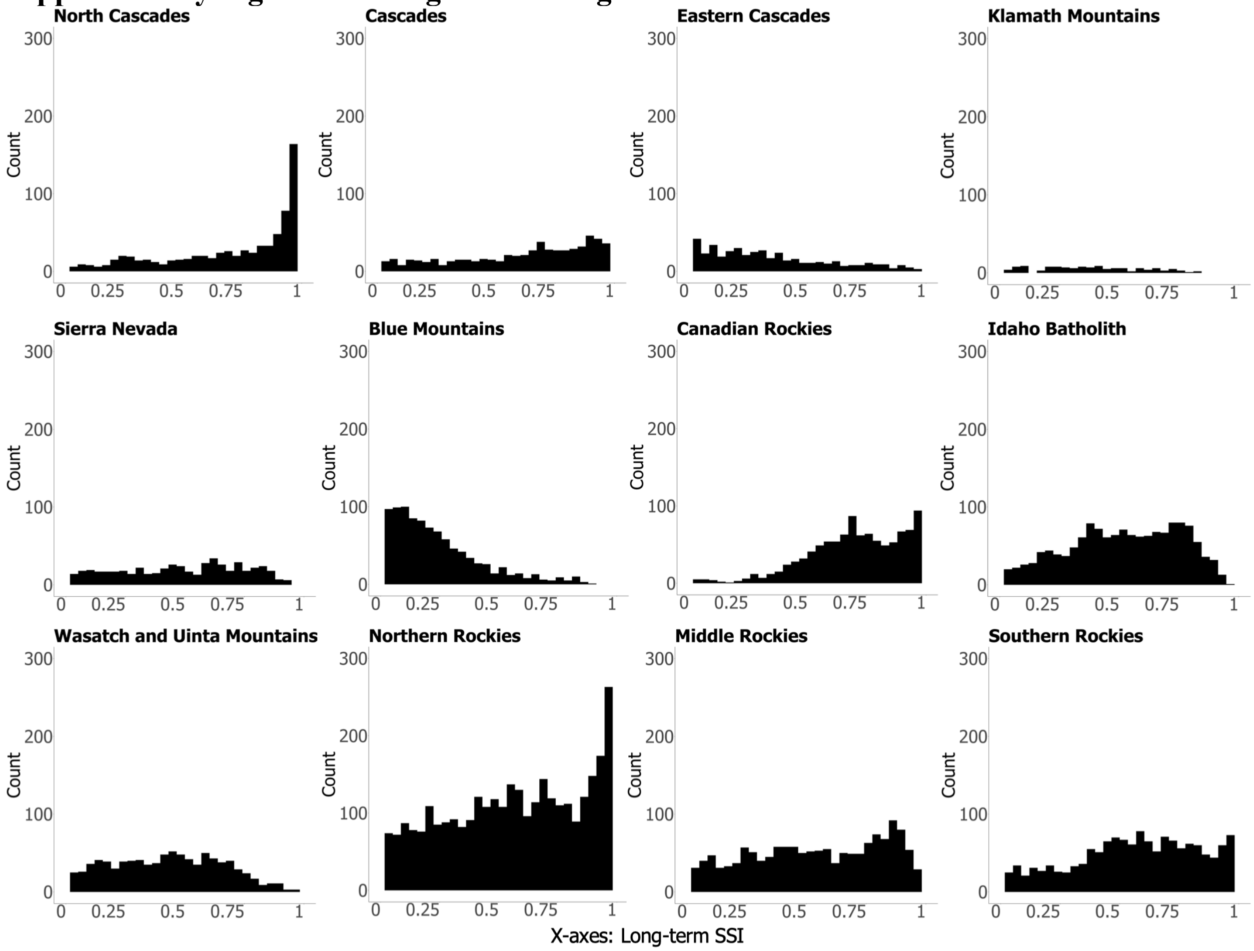

Histograms of long-term $\overline{\mathrm{SSI}}$ distribution in each ecoregion within the study domain (where $\overline{\mathrm{SSI}} \geq 0$).

Supplementary Figure 2: Weak relationships between SSI versus SWE and elevation.

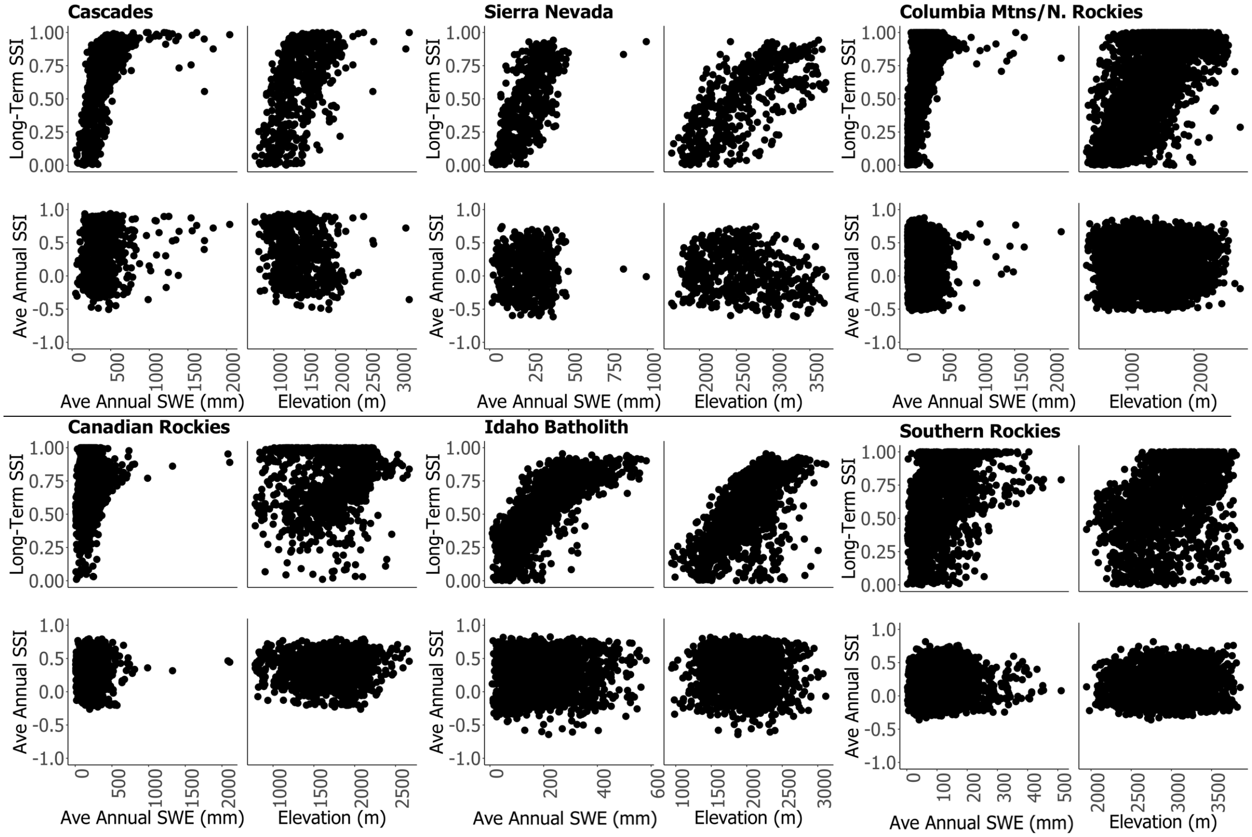

Total annual SWE (mm) and elevation (m) were compared to long-term $\overline{\text{SSI}}$ and average-annual SSI in each major ecoregion within the study domain. Relationships are either insignificant (p > 0.05) or generate $r^2 < 0.3$.

Supplementary Figure 3: Interannual variance in SSI.

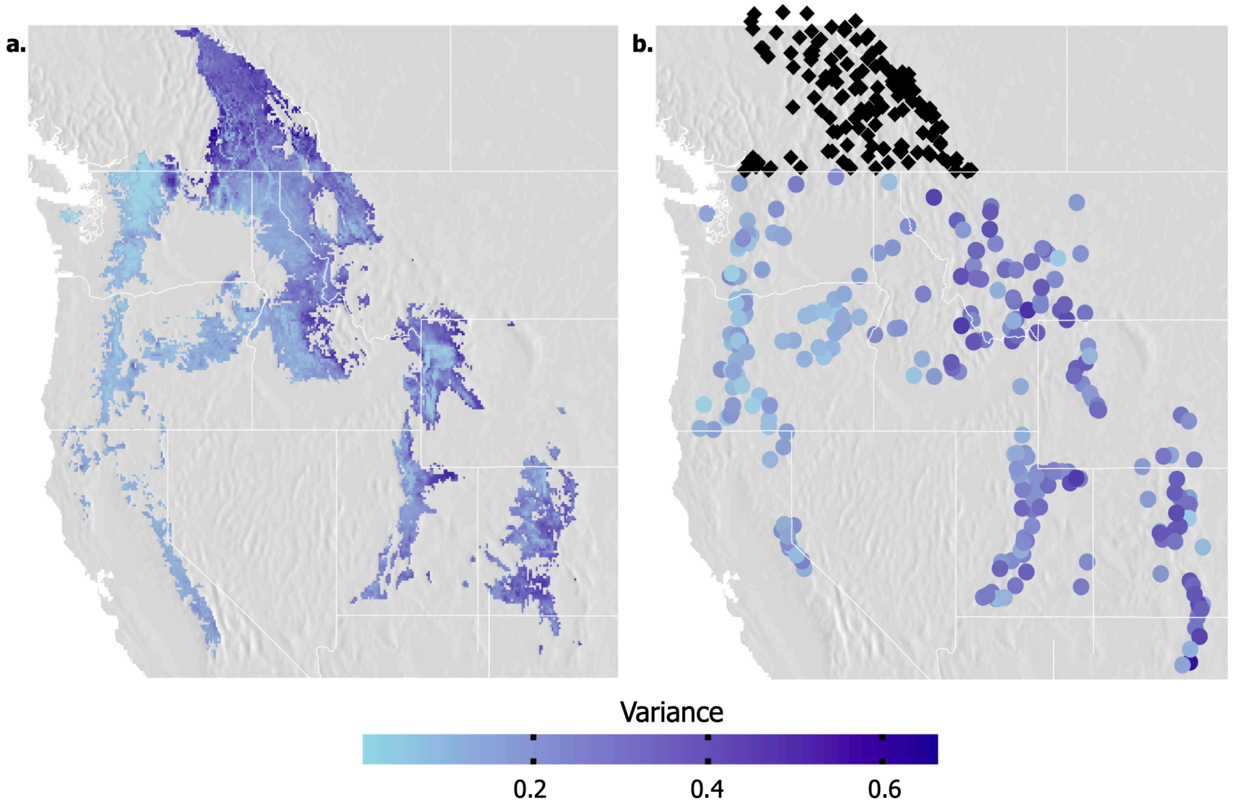

(**a**) VIC-based interannual variance in SSI (1950-2013). Panel (**b**) shows the SNOTEL-based interannual variance (1984-2018); the black diamonds represent location of the CanSWE stations, which monitor April 1 SWE.

Supplementary Figure 4: Decadal analysis of SSI trends across Western North America.

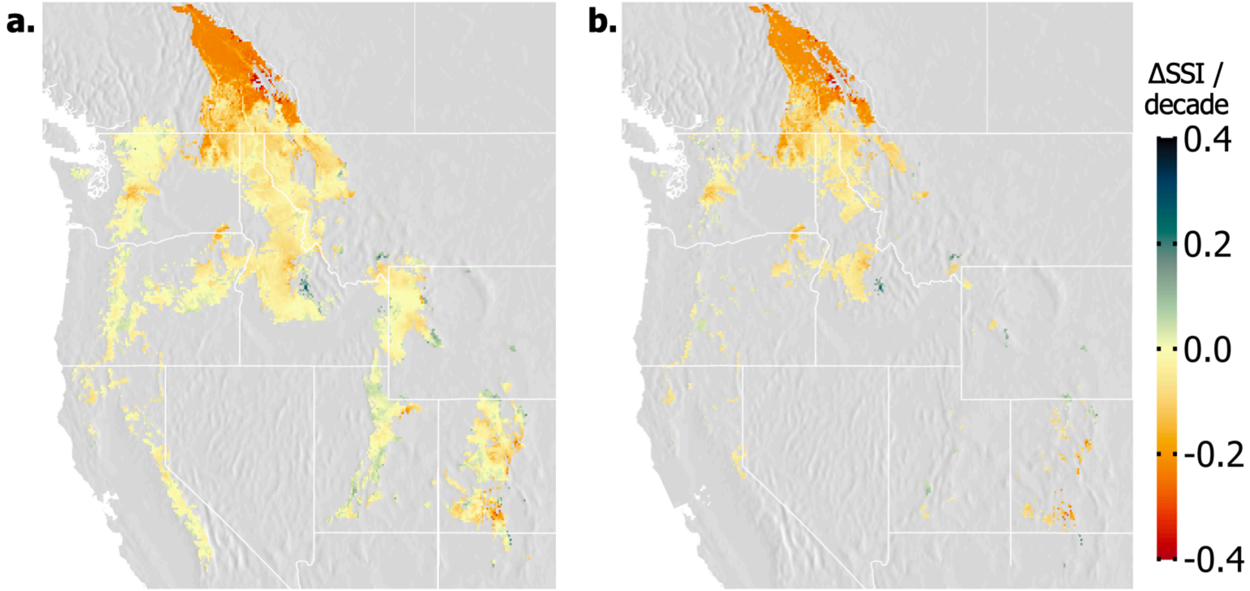

Using decade-average SSI values, (**a**) the VIC-based ΔSSI per decade (1950-2013) for all grid cells with $\overline{\text{SSI}} \geq 0$, and (**b**) for areas where the change in SSI is significant ($p < 0.05$), equating to 27% of the domain.

Supplementary Figure 5: Average annual SNOTEL-based SSI across western North America.

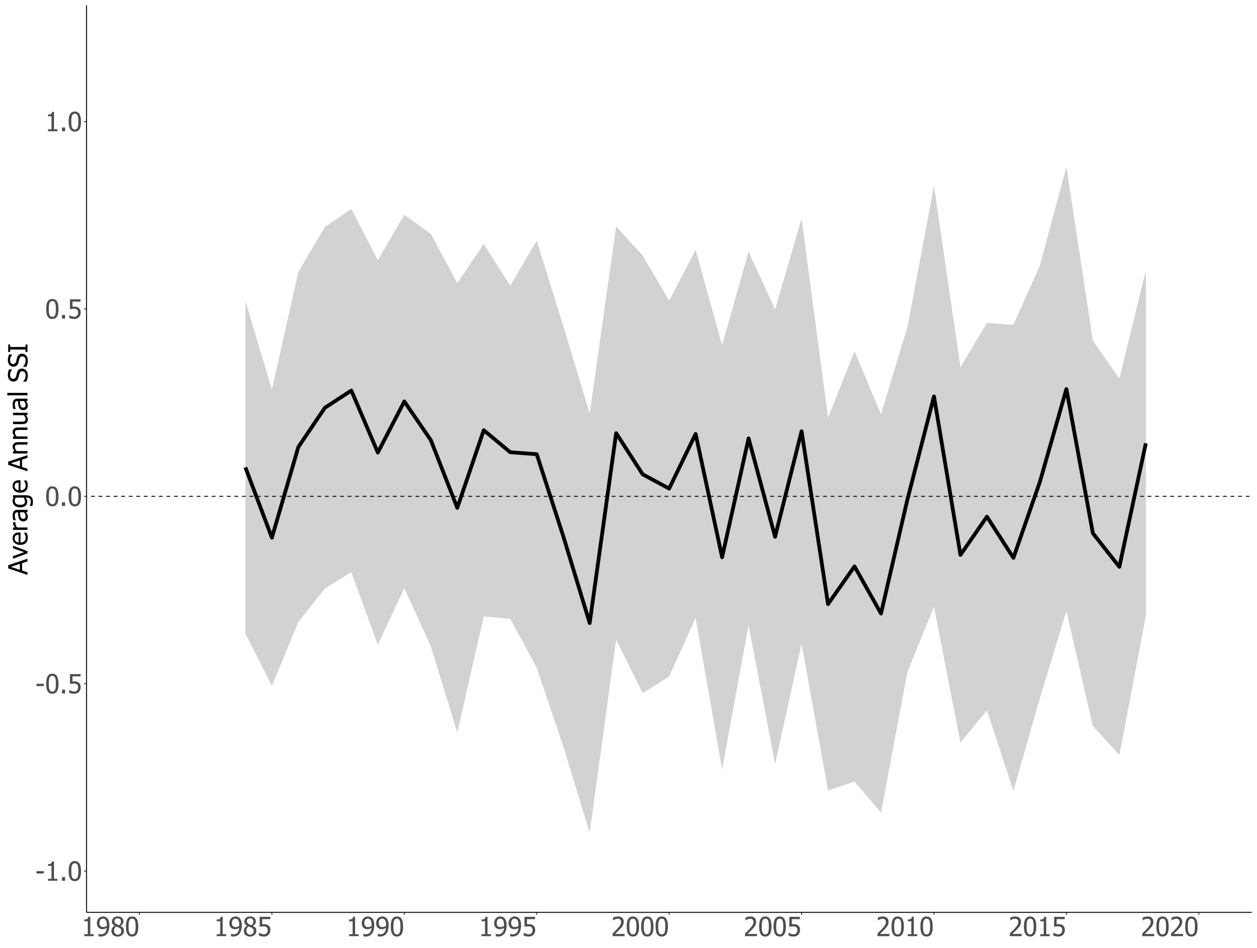

SNOTEL-based annual SSI, averaged by station after SSI was calculated (black line), standard error is shown with gray shading ($p > 0.05$).

Supplementary Figure 6: Monthly precipitation, surface water inputs, snowmelt, and rainfall in Canadian Rockies and Columbia Mountains/Northern Rockies ecoregions.

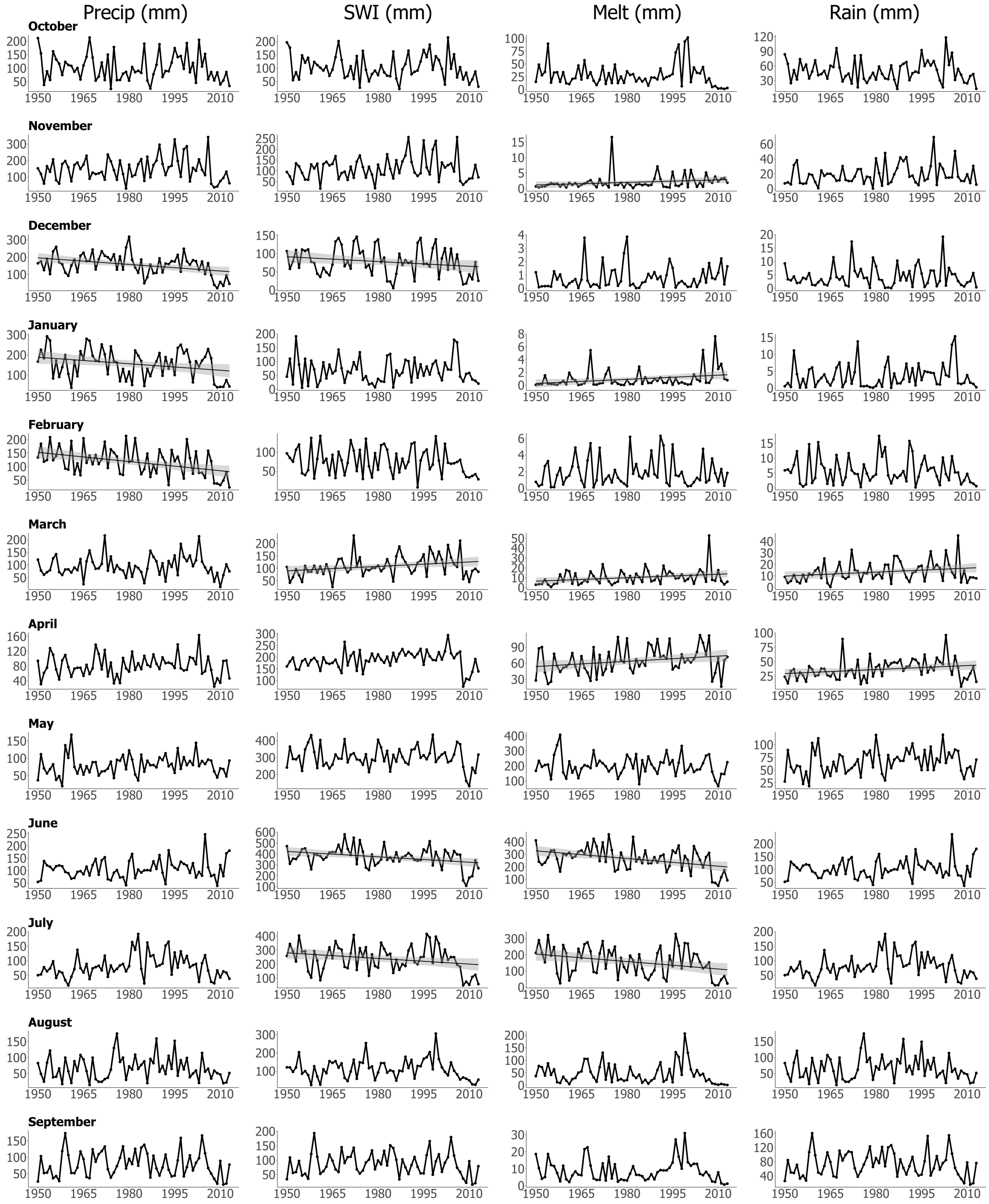

Trend lines indicate $p < 0.05$, standard error is shown with gray shading.

Supplementary Figure 7: Relationship between modeled and observed long-term SSI.

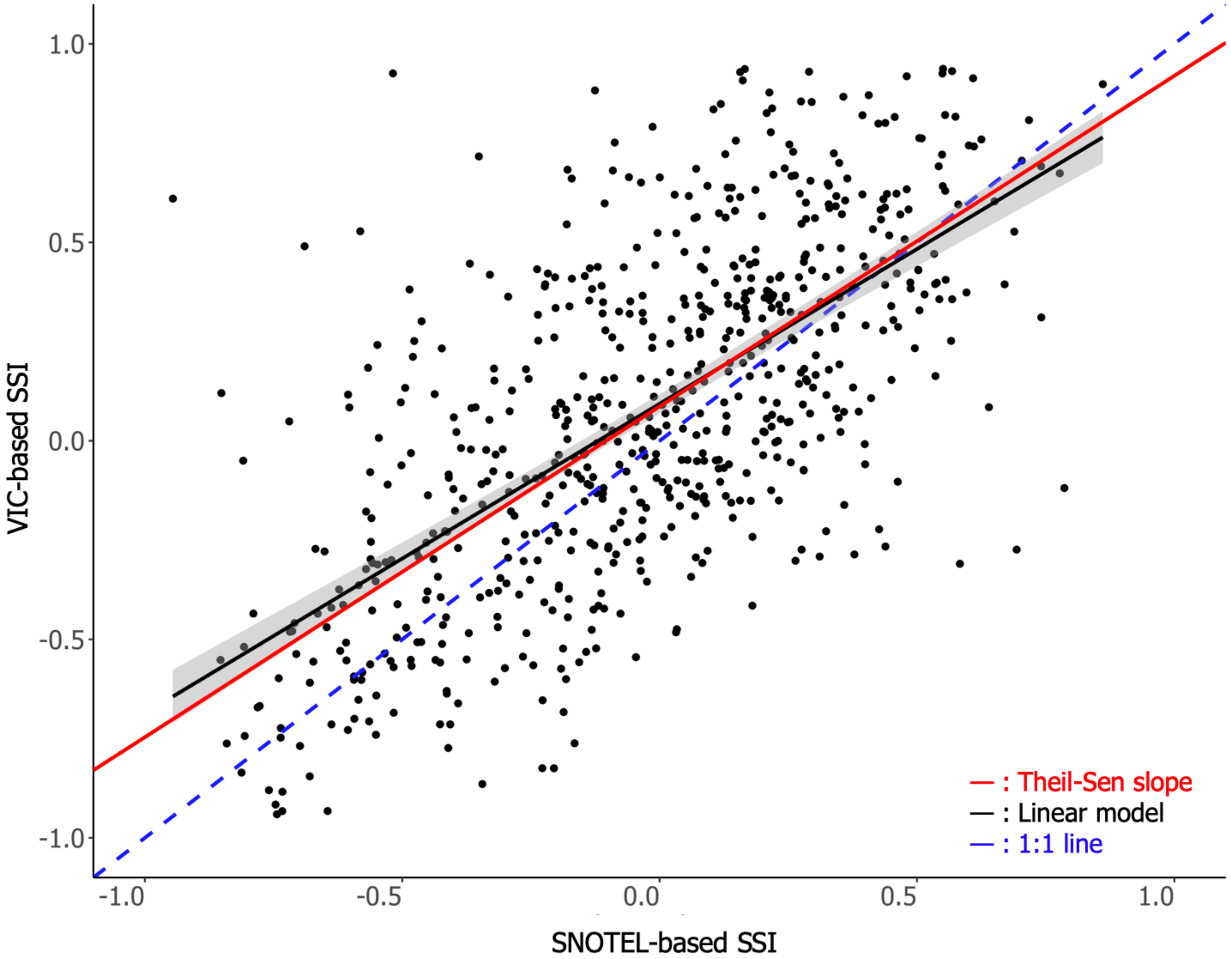

SNOTEL-based $\overline{SSI}$ vs. VIC-based modeled $\overline{SSI}$ in corresponding locations (linear model slope: 0.78, Theil-Sen slope: 0.83, r^2: 0.51).

Supplementary Figure 8: Normalized residual sum-of-squares of modeled and observed precipitation and surface water inputs data.

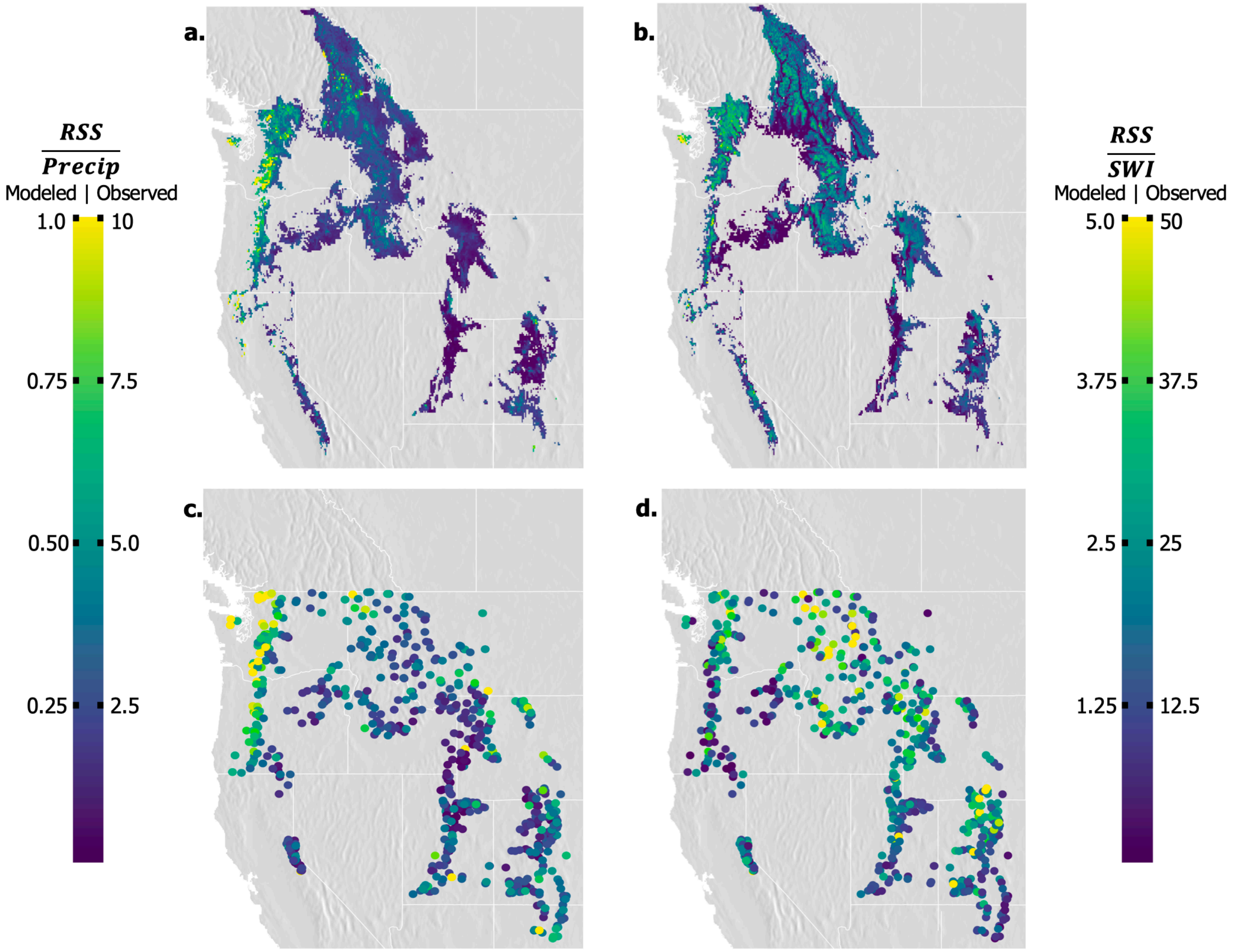

Residual sum-of-squares (RSS), normalized by (**a**) long-term VIC-based precipitation, (**b**) SNOTEL-based precipitation, (**c**) VIC-based surface water inputs (SWI), and (**d**) SNOTEL-based SWI. The RSS was calculated using daily data in millimeters. Each color bar represents the magnitude of normalized RSS for modeled (left) and observed (right).
